# The use of electronic healthcare records for colorectal cancer screening referral decisions and risk prediction model development

**DOI:** 10.1186/s12876-020-01206-1

**Published:** 2020-03-25

**Authors:** Jennifer Anne Cooper, Ronan Ryan, Nick Parsons, Chris Stinton, Tom Marshall, Sian Taylor-Phillips

**Affiliations:** 1 Division of Health Sciences, Warwick Medical School, University of Warwick, Coventry, CV4 7AL UK; 2 Institute of Applied Health Research, University of Birmingham, Birmingham, B15 2TT UK

**Keywords:** Colorectal neoplasms, Prediction model, Early detection of cancer, Mass screening, Occult blood, Electronic health records

## Abstract

**Background:**

The database used for the NHS Bowel Cancer Screening Programme (BCSP) derives participant information from primary care records. Combining predictors with FOBTs has shown to improve referral decisions and accuracy. The richer data available from GP databases could be used to complement screening referral decisions by identifying those at greatest risk of colorectal cancer. We determined the availability of data for key predictors and whether this information could be used to inform more accurate screening referral decisions.

**Methods:**

An English BCSP cohort was derived using the electronic notifications received from the BCSP database to GP records. The cohort covered a period between 13th May 2009 to 17th January 2017. Completeness of variables and univariable associations were assessed. Risk prediction models were developed using Cox regression and multivariable fractional polynomials with backwards elimination. Optimism adjusted performance metrics were reported. The sensitivity and specificity of a combined approach using the negative FOBT model plus FOBT positive patients was determined using a probability equivalent to a 3% PPV NICE guidelines level.

**Results:**

292,059 participants aged 60–74 were derived for the BCSP screening cohort. A model including the screening test result had a C-statistic of 0.860, c-slope of 0.997, and R^2^ of 0.597. A model developed for negative screening results only had a C-statistic of 0.597, c-slope of 0.940, and R^2^ of 0.062. Risk predictors included in the models included; age, sex, alcohol consumption, IBS diagnosis, family history of gastrointestinal cancer, smoking status, previous negatives and whether a GP had ordered a blood test. For the combined screening approach, sensitivity increased slightly from 53.90% (FOBT only) to 58.82% but at the expense of an increased referral rate.

**Conclusions:**

This research has identified several potential predictors for CRC in a BCSP population. A risk prediction model developed for BCSP FOBT negative patients was not clinically useful due to a low sensitivity and increased referral rate. The predictors identified in this study should be investigated in a refined algorithm combining the quantitative FIT result. Combining data from multiple sources enables fuller patient profiles using the primary care and screening database interface.

## Background

Screening for colorectal cancer (CRC) using the faecal occult blood test (FOBT) has been shown to reduce relative risk of CRC mortality by 16% in a meta-analysis of 4 randomised trials [1]. CRC screening is currently implemented in most European countries as well as parts of North and South America, Asia, Canada and Oceania [2]. Most countries use FOBTs for screening (guaiac FOBTs and faecal immunochemical tests – FITs) with organised programmes predominantly now using the FIT [2].

Currently, in England, men and women between the ages 60 to 74 are invited for bowel cancer screening biennially. The quantitative Faecal Immunochemical Test (FIT) which has shown improved accuracy and increased uptake [3] was rolled out in 2019 and is replacing the guaiac test across the screening hubs. More recently the National Screening Committee (NSC) have recommended reducing the starting age of screening to age 50, the feasibility and scheduling of this change remain to be determined. Different risk stratifying approaches can be investigated to ensure sustainability of the programme due to increased uptake and positivity of the screening test, a younger age range and the growing prevalence of CRC. This will allow limited resources such as colonoscopy to be optimised.

An approach which identifies those at greatest risk for colonoscopy services could improve the sustainability and cost-effectiveness of the screening programme, whilst reducing false positive and/or false negative results. Additional predictors along with the screening test result have been previously used to identify participants at increased risk of CRC to prioritise for referral. For instance, incorporating family history improved advanced adenoma detection [4], and combining the Asia-Pacific Colorectal Screening score with the screening test result helped to identify higher risk groups for referral [5]. Risk prediction models have also been developed which combine the screening test with other risk factors for individualised prediction leading to an improvement in sensitivity [6, 7].

Combining lab test results with FOBTs has also been shown to improve the detection of cancer [8, 9]. A FOBT on its own, without other predictors may fail to detect intermittent bleeding or smaller lesions which may not bleed. Routine blood test results have been used to predict risk of CRC for use in screening by generating a risk score [10]. Systematic reviews have identified improved test performance when combining faecal and serum biomarkers or FOBTs with blood test results [11, 12]. Studies which have developed risk prediction models or identified symptoms and diagnostic features of CRC in a primary care setting have also been investigated [13–15]. The QCancer risk prediction model was developed to prioritise patients at sufficient risk for primary care referral. External validation of the discrimination of the model gave an AUC of 0.91 for men and 0.92 for women [15, 16].

Electronic health record data is increasingly used for research. Initiatives are underway to link disparate datasets across health services to derive further insight for patient care and to enable smarter use of limited resources/services. Combining data from multiple sources allows a clearer and fuller picture of patient profiles and their interactions with different healthcare services.

A model exploiting the data interface between primary care and screening data systems for use in a screening population has not previously been investigated. The richer data available from GP databases could be used to add a further dimension to a CRC screening model to improve discriminatory power and referral decisions.

The aims of this study using The Health Improvement Network (THIN database) were to: (i) identify predictors of CRC and polyps for a BCSP population and their completeness ii) determine the risk of CRC/polyps for these clinical features for a BCSP population (iii) develop multivariable risk prediction models using predictors derived from both the BCSS and from GP Records and whether these models could be used to inform more accurate screening referral.

## Methods

The following reporting guidelines were used; Reporting of studies Conducted using Observational Routinely collected Data (RECORD) [17], and the Transparent Reporting of a multivariable prediction model for Individual Prognosis Or Diagnosis (TRIPOD) [18].

### Source of data

The Health Improvement Network (THIN) database of anonymised GP records was used for analysis and has data for over 17 million patients in the UK (with 3.1 million active patients and > 5% coverage) [19]. THIN includes primary care practices which use Vision software and provides demographic information such as sex, age, Townsend deprivation score, diagnoses, symptoms and prescriptions.

The Bowel Cancer Screening System (BCSS) used in the NHS Bowel Cancer Screening Programme (BCSP) is used to identify participants and record test results. There are interconnections between the BCSS and primary care records. The BCSS receives its data originally from GP records for its participants in the relevant age range (through upload to the NHS Information Authority and the NHS Spine). Since 2009–2010 GP practice systems have been able to opt into receiving electronic screening results from the BCSS using the same system as the Pathology Messaging Implementation Programme (PMIP).

An English BCSP cohort was derived using the electronic notifications received from the Bowel Cancer Screening System to GP records. THIN was used to derive this cohort by identifying men and women with automatically received electronic notifications from the BCSP, aged 60–74 years of age and with at least a years’ worth of health records before taking their latest FOBT (to ensure adequate symptomatic information to be identified). This covered a period between 13th May 2009 (the first FOBT screen date) with follow up to 17th January 2017 (the last follow up date). Patients were excluded if they had a previous CRC diagnosis or if they had a high-risk condition (hereditary nonpolyposis colorectal cancer – HNPCC) or familial adenomatous polyposis (FAP)).

Practice eligibility used the latest of the following: one year after the Vision practice software installation, the acceptable mortality recording (AMR) date [20] and the date in which the electronic BCSP notifications started to be received by the practice (the full details of defining this date for each practice will be published elsewhere). Before electronic notifications were received, data may be incomplete, subject to transcription errors or biased towards positive results.

### Predictors

Predictors investigated were taken from the interface between the BCSS (previous positive or negative screening results) and GP records (demographics, lifestyle factors, anthropometrics, laboratory test results, symptoms present within the screening population) and were derived from previous research and NICE guidelines [13, 21–24].

All previous BCSP FOBT results were extracted in order to have an individual’s screening history and originated from the BCSS. Predictors were derived from the GP database using Read code lists (Read Version 2) for 28 clinical features. Clinical lists developed were subject to a double reviewing process for code set validation.

Last recorded entry was used for the following variables: smoking status, alcohol consumption and family history. The TRIPOD guidelines recommend using a continuous variable rather than dichotomising into different groups as this loses additional predictive information [25]. Cut-offs for certain blood tests are employed in clinical practice since it can indicate underlying disease, therefore categorised blood measurements were also considered for: platelet count, ferritin, haemoglobin concentration and mean cell volume. Variables assessed for univariable and multivariable analysis and how they were operationalised are provided in Supplementary Table S1.

Studies have suggested that large proportions of colorectal cancer screening participants have underlying symptoms [26–28] despite recommendations and campaigns for symptomatic individuals to visit their GP. Some of these symptoms can be considered ‘low risk, but not no risk’ [29] and are often self-limiting but in combination can indicate underlying disease [13, 14]. Symptoms present within the screening cohort were measured at the time of entry to the study up to 365.25 days before the index date. Drug code lists were generated for 3 types of prescriptions; anti-motility drugs, antispasmodics and laxatives using the British National Formulary and key word searches. Prescriptions were investigated as a proxy to a particular clinical feature as performed in previous research by the authors [13].

### Outcome

The index date used for survival analysis was the date of the latest BCSP FOBT result. The outcome was a diagnosis of CRC/polyps up to 2 years after the index date (latest FOBT) recorded in a patient’s record. Two years represents one screening round in the NHS and allows for the clinical identification of interval cancers. The earliest date of diagnosis was used if both polyps and CRCs had been diagnosed within the 2-year follow up.

### Sample size

For stable predictions it has been recommended that multivariable models include at least 10 outcome events per degree of freedom [18]. The dataset for multivariable modelling analysis had 1676 CRC and polyp diagnoses and considered 17 degrees of freedom giving 98.59 outcomes per degree of freedom. The dataset for the model with negative FOBTs only included 735 outcome events and considered 16 degrees of freedom giving 45.94 outcomes per degree of freedom.

### Statistical analysis

#### Overview

To identify predictors for CRC/polyps in a BCSP population, the proportion of individuals with particular clinical features was assessed along with the completeness of data. The level of complete/missing data was recorded in order to determine the availability of predictors from primary care records which could contribute to referral algorithms. The risk of CRC/polyps for these 28 clinical features in a screening population was assessed using univariable Cox regression to estimate hazard ratios.

Two risk prediction models were developed (and internally validated) using Cox Regression with a diagnosis of CRC/polyp recorded in a patient’s record as the outcome. For model development, those with red flag symptoms which includes those defined by NICE guidelines for suspected cancer referral were excluded (rectal bleeding, abdominal mass, abnormal rectal exam, change in bowel habit, abdominal pain, weight loss, iron deficiency anaemia (haemoglobin < 12 g/dL for females < 13 g/dL for men, ferritin < 15 μg/L and MCV < 80 fL). In addition, those with a diagnosis of previous polyps or an FOBT result ordered through primary care were excluded.

The first model used a population with both positive and negative FOBT results to determine the absolute probability of CRC for someone who has taken a screening test. This approach could be used to prioritise screening referrals to colonoscopy for those at highest risk. The second model included only patients with a negative FOBT to determine whether other factors could be used to decide whether a person is at sufficient risk to be referred despite a negative result.

Absolute risk predictions were determined from the models for each patient and their personal predictors (covariate pattern). The negative model was applied to a subset of the population who had complete data and 2 year follow up (*n* = 25,592). A predetermined risk probability cut-off which represents the NICE guidelines risk level of 3% [21], was used for those with a negative result. Test accuracy of the FOBT alone was compared to a strategy of combining the model positives with FOBT positives (sensitivity, specificity, PPV, NPV reported). The number of extra participants who would need lower gastrointestinal (GI) investigations and number of extra polyps/cancers were determined.

Cox regression (time-to-event) was employed over logistic regression due to the longitudinal nature of the data. Individuals have different lengths of follow up on the database (i.e. reach the study end before the outcome occurs, move GP practices, death etc). Patients who are right-censored in this way provide valuable information up to their final point of follow up [30]. Employing survival models is a more efficient use of the data by maximising events at the tail end. Furthermore, the predictions for these models are over a period of two years and it is argued that predictions for time periods over 6 months should consider time-to-event regression modelling [30]. Similar studies using electronic health records for model development and validation in a primary care setting have also used survival analysis aiding comparability of the model in a screening context [15, 16].

#### Model development

Analyses used Stata SE Version 15.1. Cox regression and multivariable fractional polynomials with backwards elimination was used to develop each model using the ‘mfp’ function in Stata [31, 32]. Age at FOBT and sex were forced into the models due to clinical relevance. Multivariable fractional polynomials (MFPs) allow non-linear relationships with continuous predictors to be modelled [32]. For backwards elimination, a *p*-value of 0.05 was used to determine whether to keep a predictor in the model (a variable is removed if dropping it from the model causes a non-significant increase in the deviance) [32]. *P*-values for testing between fractional polynomial models and for assessing interactions was set at 0.05. Interactions included: age and sex, FOBT result and sex, FOBT result and smoking, smoking and sex. When reporting the final model, the Cox Regression coefficients are provided along with bootstrapped standard errors (100 bootstrap replications due to model complexity and size).

Multiple imputation was considered for missing data however the missing data mechanism for the majority of these predictors would be ‘Missing not at random’ (MNAR), consequently complete cases were used for these analyses. For the multivariable models, alcohol consumption was the predictor which limited the sample size (78% recorded for the derived screening cohort). Other variables such as BMI (95.85%) and smoking status (99.44%) were highly complete.

#### Model performance

The model performance was assessed using Harrell’s C statistic (to measure discrimination or how well predictions separate those with and without the outcome). Calibration of the models was assessed by plotting a calibration curve for the models once adjusted for optimism. Other performance measures assessed included Somers’ D rank correlation (D = 2(C-0.5)) which ranges from − 1 to 1 [33, 34], the D statistic, R^2^ and adjusted R^2^.

The optimism of the models was assessed by calculating the heuristic shrinkage factor of Van Houwelingen [35]. To adjust performance statistics for optimism, internal validation was performed using 100 bootstrap replications for the C statistic, c-slope, D statistic and R^2^. A split sample approach to model development is generally not recommended; bootstrap validation for assessing statistical optimism is preferred, although less of an issue for large sample sizes with sufficient events and lower model complexity [18].

#### Absolute risk predictions

Predicted probabilities of CRC/polyps were derived for each patient and their covariate pattern. The baseline CRC free survival was combined with the linear predictor to generate individualised predictions. The full risk equations are provided for both the models.

Non-parametric estimation of the CRC free survival was obtained using a zero covariate value and the methods implemented in Stata. CRC free survival for two years was obtained from the Kaplan-Meier curve and accompanying results. The shrunken linear predictor was used to estimate a new baseline CRC free survival (adjusted for optimism) which was estimated non-parametrically at 2 years. The shrunken linear predictor was combined with the baseline CRC free survival to generate risk predictions. In order to obtain an event probability, the result of this was subtracted from 1 to generate the probability of CRC/polyps being diagnosed over a 2 year period.

#### Clinical implications

The prediction model developed for those with negative FOBTs could be used to increase the low sensitivity of screening [36] by identifying additional patients for referral based on a combination of symptoms and demographic characteristics. The negative FOBT model was applied to a subset of the population who had complete data and 2 year follow up (*n* = 25,592). Individualised probabilities for CRC/polyps were determined from the model and an appropriate threshold applied for referral. A predetermined probability cut-off (0.0168) which corresponds to the NICE guidelines PPV risk level of 3% [21], was used for those with a negative result (*n* = 24,297). This was determined by plotting PPV and NPV against different risk probability cut-offs. The ROC curve for this model was generated and the test characteristics (sensitivity, specificity and NPV) reported. The number of extra participants who would need lower gastrointestinal (GI) investigations and number of extra polyps/cancers were determined.

## Results

### Study population

The screened cohort included 292,059 patients across 360 practices aged 60–74 with 6362 positive and 285,697 negative FOBTs (2.2% test positive). The cohort was 53.26% female, with a mean age of 66.43. The earliest diagnosis in 2 years was CRC for 849 patients and polyps for 2040 patients (2889 total). The study flow diagrams for both data extraction and for deriving the screening cohort from THIN are presented in Supplementary Figs. S1 and S2. Test accuracy was measured for a population with a minimum of 2 years follow up (*n* = 30,187, screening test positivity 5.41%). The two year sensitivity for the guaiac FOBT was 51.21% and specificity 96.28% and is similar to reported values in the literature [36, 37]. The two by two table is provided in Supplementary Table S2.

### Completeness of records

The completeness of variables in the cohort of patients aged 60–74 with a FOBT result is summarised in Table 1. Age, sex and GP practice were complete, ethnicity was present in 54.76%, smoking status was present in 99.44%, alcohol consumption in units per week in 78.00% and BMI in 95.85%.

**Table 1:** Variable completeness and univariable associations with colorectal cancer and polyps

Variable	Percentage with this variable recorded (*N* = 292,059)		Prevalence of variable (%)		Hazard Ratio (95% Confidence Interval)		Standard Error	*P* > z
**Sociodemographic characteristics**								
Sex	100							
Male (baseline)	–		46.74%	(136,518/292,059)	–	(---)	–	–
Female	–		53.26%	(155,541/292,059)	0.655	(0.609–0.706)	0.025	0.000*
Age at Latest FOBT (continuous)	100		Mean 66.43 (SD 4.47)		1.025	(1.017–1.033)	0.004	0.000*
**BCSP Screening History (initially derived from BCSS)**								
**Latest FOBT Result (initially derived from BCSS)**	100	(292,059/292,059)						
BCSP FOB test normal (baseline)	97.82	(285,697/292,059)	97.82%	(285,697/292,059)	–	(---)	–	–
BCSP FOB test abnormal	2.18	(6362/292,059)	2.18%	(6362/292,059)	55.936	(51.988–60.183)	2.089	0.000*
Previous Positive BCSP FOBTs (continuous)		(Recorded if observed)	0 (99.47%) 1 (0.51%) 2 (0.02%) 3 (0.00068%)	(290,515/292,059) (1488/292,059) (54/292,059) (2/292,059)	5.028	(4.180–6.047)	0.473	0.000*
Previous Negative BCSP FOBTs (continuous)		(Recorded if observed)	0 (60.44%) 1 (31.18%) 2 (8.03%) 3 (0.34%) 4 (0.0027%)	(176,523/292,059) (91,076/292,059) (23,465/292,059) (987/292,059) (8/292,059)	0.769	(0.720–0.821)	0.026	0.000*
Previously screened with a BCSP FOBT		(Recorded if observed)	39.94% with 60.06% without	(116,641/292,059) (175,418/292,059)	0.783	(0.723–0.847)	0.032	0.000*
**Lifestyle characteristics and measurements**								
Alcohol (units per week) (continuous)	78.00	(227,792/292,059)	Mean 9.49 (SD 12.27)		1.010	(1.008–1.011)	0.001	0.000*
**Smoking Status**	99.44	(290,429/292,059)						
Never-Smoked	57.48	(167,880/292,059)	57.80%	(167,880/290,429)				
Ex-Smoker	33.32	(97,310/292,059)	33.51%	(97,310/290,429)	1.532	(1.417–1.656)	0.061	0.000*
Current Smoker	8.64	(25,239/292,059)	8.69%	(25,239/290,429)	1.619	(1.437–1.824)	0.099	0.000*
**Anthropometrics**								
BMI (continuous)	95.85	(279,927/292,059)	Mean 27.48 (SD 5.01)		1.029	(1.022–1.036)	0.004	0.000*
**Laboratory test results**								
Primary care FOBT		(Recorded if observed)	0.01% with 99.99% without	(32/292,059) (292,027/292,059)	2.868	(0.404–20.369)	2.869	0.292
Hb g/dL (continuous) within 365 days prior to the latest FOBT	44.51	(129,996/292,059)	Mean 13.92 (SD 1.30)		0.990	(0.953–1.029)	0.019	0.606
Hb < 11 g/dL (reference category ≥11 g/dL) within 365 days prior to the latest FOBT	44.51	(129,996/292,059)	1.50% < 11 g/dL 98.50% ≥11 g/dL	(1947/129,996) (128,049/129,996)	2.231	(1.679–2.966)	0.324	0.000*
Mean Cell Volume fL (continuous) within 365 days prior to the latest FOBT	44.33	(129,481/292,059)	Mean 91.11 (SD 5.08)		0.996	(0.986–1.005)	0.005	0.382
Mean Cell Volume < 80 fL (reference category ≥80 fL) within 365 days prior to the latest FOBT	44.33	(129,481/292,059)	1.60% < 80 fL 98.40% ≥80 fL	(2073/129,481) (127,408/129,481)	2.419	(1.856–3.151)	0.326	0.000*
Ferritin 15 μg/L (continuous) within 365 days prior to the latest FOBT	8.59	(25,082/292,059)	Mean 127.07 (SD 201.66)		0.999	(0.998–1.000)	0.000	0.069
Ferritin < 15 μg/L (reference category ≥15 μg/L) within 365 days prior to the latest FOBT	8.59	(25,082/292,059)	4.99% < 15 μg/L 95.01% ≥15 μg/L	(1252/25,082) (23,830/25,082)	2.054	(1.434–2.943)	0.377	0.000*
Platelet Count × 10^9^/L (continuous) within 365 days prior to the latest FOBT	44.40	(129,685/292,059)	Mean 245.61 (SD 66.00)		1.000	(0.999–1.001)	0.000	0.691
Platelet Count > 400 × 10^9^/L (reference category ≤400 10^9^/L) within 365 days prior to the latest FOBT	44.40	(129,685/292,059)	2.13% > 400 × 10^9^/L) 97.87% ≤400 10^9^/L	(2764/129,685) (126,921/129,685)	1.155	(0.837–1.594)	0.190	0.379
GP has ordered a blood test 365 days prior to their latest BCSP FOBT		(Recorded if observed)	44.72% with 55.28% without	(130,611/292,059) (161,448/292,059)	1.441	(1.339–1.550)	0.054	0.000*
**Other Conditions/Diagnoses**								
Previous polyps diagnosed		(Recorded if observed)	2.49% with 97.51% without	(7269/292,059) (284,790/292,059)	3.181	(2.767–3.658)	0.226	0.000*
Diabetes		(Recorded if observed)	11.05% with 88.95% without	(32,272/292,059) (259,787/292,059)	1.470	(1.329–1.627)	0.076	0.000*
Crohn’s disease		(Recorded if observed)	0.30% with 99.70% without	(884/292,059) (291,175/292,059)	1.038	(0.539–1.997)	0.346	0.911
Ulcerative Colitis		(Recorded if observed)	0.61% with 99.39% without	(1796/292,059) (290,263/292,059)	1.686	(1.177–2.416)	0.309	0.004*
Irritable Bowel Syndrome		(Recorded if observed)	9.28% with 90.72% without	(27,103/292,059) (264,956/292,059)	1.141	(1.013–1.286)	0.069	0.030*
Diverticulitis		(Recorded if observed)	6.37% with 93.63% without	(18,606/292,059) (273,453/292,059)	1.226	(1.069–1.406)	0.086	0.004*
Venous Thromboembolism		(Recorded if observed)	0.31% with 99.69% without	(916/292,059) (291,143/292,059)	1.421	(0.824–2.451)	0.395	0.206
Family History of Gastro-Intestinal Cancer		(Recorded if observed)	1.51% with 98.49% without	(4423/292,059) (287,636/292,059)	1.591	(1.251, 2.023)	0.195	0.000*
**GP recorded Symptoms**								
Constipation		(Recorded if observed)	1.46% with, 98.54% without	(4260/292,059) (287,799/292,059)	1.654	(1.305–2.097)	0.200	0.000*
Diarrhoea		(Recorded if observed)	2.01% with, 97.99% without	(5867/292,059) (286,192/292,059)	1.779	(1.464–2.161)	0.177	0.000*
Loss of Appetite		(Recorded if observed)	0.04% with, 99.96% without	(117/292,059) (291,942/292,059)	2.614	(0.843–8.109)	1.510	0.096
Flatulence		(Recorded if observed)	0.17% with 99.83% without	(498/292,059) (291,561/292,059)	2.481	(1.439–4.278)	0.670	0.001*
Tiredness		(Recorded if observed)	2.46% with 97.54% without	(7173/292,059) (284,886/292,059)	1.358	(1.108–1.665)	0.141	0.003*
Weight Loss		(Recorded if observed)	0.36% with 99.64% without	(1057/292,059) (291,002/292,059)	1.705	(1.073–2.710)	0.403	0.024*
Change in Bowel Habit		(Recorded if observed)	0.57% with, 99.43% without	(1655/292,059) (290,404/292,059)	2.610	(1.924–3.539)	0.406	0.000*
Abdominal Pain†		(Recorded if observed)	7.12% with, 92.88% without	(20,790/292,059) (271,269/292,059)	1.425	(1.261–1.610)	0.089	0.000*
Abdominal Pain		(Recorded if observed)	4.86% with 95.14% without	(14,206/292,059) (277,853/292,059)	1.424	(1.232–1.646)	0.105	0.000*
Abdominal Mass		(Recorded if observed)	0.06% with 99.94% without	(165/292,059) (291,894/292,059)	1.258	(0.314–5.032)	0.890	0.746
Rectal Bleeding/melaena		(Recorded if observed)	0.92% with 99.08% without	(2694/292,059) (289,365/292,059)	3.118	(2.504–3.884)	0.349	0.000*
**Drug Prescriptions**								
Antispasmodic drug prescription		(Recorded if observed)	3.31% with 96.69% without	(9661/292,0590 (282,398/292,059)	1.450	(1.221–1.721)	0.127	0.000*
Anti-motility drug prescription		(Recorded if observed)	1.24% with 98.76% without	(3613/292,0590 (288,446/292,059)	1.535	(1.176–2.005)	0.209	0.002*
Laxative Drug		(Recorded if observed)	7.96% with 92.04% without	(23,234/292,059) (268,825/292,059)	1.390	(1.235–1.564)	0.084	0.000*

* Significant at the *p* value of 0.05

† Includes prescriptions of anti-spasmodic drug.

*FOBT* Faecal Occult Blood Test, *BCSP* bowel cancer screening programme, *BMI* body mass index, *MCV* mean cell volume, *IBS* irritable bowel syndrome, *Hb* haemoglobin concentration

Full blood count results were present in around 45% of patients (for Hb, MCV and platelet count) whereas ferritin was present for 8.59%. The cancer/polyp detection rate for those with a laboratory record (for all three results) was around 1.19% and those without 0.83% (Pearson’s chi-squared p = < 0.001) (see Supplementary Table S3). Since the ordering of a blood test by the GP (as a clinical process) is predictive of colorectal cancer, this predictor was included in the multivariable model.

Although Quality Outcomes Framework (QOF) indicators have been introduced for recording ethnic group, this factor had 54.76% recording. Ethnic group records have a low level of recording in primary care databases [38] and there is evidence to suggest that it is currently not representative of the UK population and so this parameter was not used for multivariable analysis. The proportion of the screening cohort with the presence of one or more lower risk symptoms (diarrhoea, constipation, loss of appetite, flatulence, tiredness) was 5.84%. 8.17% (520/6362) for those with positive FOBTs and 5.79% (16,533/285,697) for those with negative FOBTs. Further considered predictors are included in Supplementary Table S4.

### Univariable associations

To determine the predictors with an association for CRC/polyps which could be used to assist referral decisions or included in a risk prediction model, the univariable hazard ratios estimated using Cox Regression are presented for the variables of interest in Table 1.

Predictors derived from the BCSP included previous positive FOBT results (HR: 5.028, CI: 4.180–6.047) previous negative FOBT results (HR: 0.769, CI: 0.720–0.821) and whether a participant had been previously screened (HR: 0.783, CI: 0.723–0.847). Lifestyle factors/anthropometrics available from GP records included alcohol consumption units per week (HR: 1.010, CI: 1.008–1.011), smoking status (HR: 1.619, CI: 1.437–1.824, for a current smoker) and BMI (HR: 1.029, CI: 1.022–1.036). Of the blood test results sent by pathology to GP records; haemoglobin, ferritin and MCV had a significant effect on the diagnosis of CRC/polyps with HRs of 2 and above when investigated with a clinical cut-point reflecting the underlying clinical pathway. If a GP had ordered a blood test result in the 365 days prior to the latest FOBT result this had a positive association for colorectal cancer (HR: 1.441, CI: 1.339–1.550). Females were at lower risk of CRC/polyp diagnosis than males (HR: 0.655, CI: 0.609–0.706). Conditions which had a positive association included diabetes (HR: 1.470, CI: 1.329–1.627) and IBS (HR: 1.141, CI: 1.013–1.286). If an individual had reported a family history of gastro-intestinal cancer the hazard ratio was 1.591 (CI: 1.339–1.550). A reported lower risk symptom such as constipation and diarrhoea were also significant predictors.

### Model populations

For multivariable analysis and patients with both positive and negative FOBTs, there were 191,081 complete cases, mean age was 66.39 years and 50.36% were female. There were 1676 outcome events, 514 CRCs and 1162 polyps. Follow up was for a total of 73,987,747.5 person days. Patients with just negative FOBT results recorded as their latest screening test result used 187,470 complete cases, mean age was 66.97 and 50.31% were female. There were 735 outcome events, 225 CRC and 510 polyps. Follow up was for a total of 72,769,587.5 days.

### Model development

The variables included in model development were the following: FOBT result, smoking status, BMI, diabetes, alcohol consumption, age at FOBT, sex, Townsend quintile, previous positive BCSP FOBTs, previous negative BCSP FOBTs, whether the GP had ordered a blood test, family history of gastro-intestinal cancer and IBS.

The final multivariable model for those with positive and negative FOBT results included: FOBT result, smoking status (ex or current smoker compared to non-smoker as reference category), alcohol consumption (units per week), sex age, previous negative FOBTs, and family history of gastro-intestinal cancer. There were no significant interactions. Alcohol consumption and age were modelled using non-linear functions selected by the MFP algorithm and previous negative results was centred. The final model is reported below with further model performance metrics in Table 2.

**Table 2:** Cox regression multivariable prediction model for participants with a FOBT result (either positive or negative) *N* = 191,081, 1676 events

Variable	Hazard Ratio	Observed Coefficient	Bootstrapped Standard Error	z	***P*** > z	[95% Confidence Intervals]	
**FOBT Result Positive (reference category negative FOBT result)**	70.173	4.251	0.057	74.19	<0.001	4.139	4.363
**Smoking Status**							
Ex-smoker (reference category non-smoker)	1.141	0.132	0.050	2.61	0.009	0.033	0.230
Current smoker (reference category non-smoker)	1.265	0.235	0.090	2.61	0.009	0.058	0.411
**((Alcohol + 1)/100)**^2^*	−	3.147	1.180	2.67	0.008	0.835	5.460
**((Alcohol + 1)/100)**^3^*	−	−4.177	1.557	−2.68	0.007	−7.229	−1.125
**Sex Female (reference category male)**	0.850	−0.162	0.054	−2.99	0.003	−0.269	−0.056
Age/10 *	−	5.859	2.064	2.84	0.005	1.814	9.904
**(Age/10)**^2^*	−	−0.419	0.154	−2.71	0.007	−0.722	−0.116
Previous Negative BCSP FOBTs*	0.862	−0.149	0.049	−3.05	0.002	−0.245	−0.053
**Family History of Gastrointestinal Cancer**	1.560	0.444	0.168	2.64	0.008	0.115	0.774

*Abbreviations: CI confidence intervals, FOBT faecal occult blood test (specifically guaiac). The continuous variables (Age/10) has been centred at 6.639, (Age/10)*^*2*^*at 44.077, ((Alcohol + 1)/100)*^*2*^*at 0.011, ((Alcohol + 1)/100)*^*3*^at 0.001, Previous negative BCSP FOBTs at 0.507. A ‘*’ indicates that the variable is treated as continuous.

***Survival Probability***

$$ S(2)={0.9932}^{\exp \left(4.25{x}_1+0.13{x}_2+0.23{x}_3+3.15\left(\ {\left(\frac{x_4+1}{100}\right)}^2-0.011\right)-4.18\left(\ {\left(\frac{x_4+1}{100}\right)}^3-0.001\right)-0.16{x}_5+5.86\left(\frac{x_6}{10}-6.639\right)-0.42\left(\ {\left(\frac{x_6}{10}\right)}^2-44.077\right)-0.15\left({x}_7-0.507\right)+0.44{x}_8\right)} $$

0.9932  baseline CRC free survival at 2 years S_0_(2) (the re-estimated shrunken baseline CRC free survival at 2 years was also 0.9932 when rounded) the heuristic shrinkage factor was 0.998.

Where S(2) is the survival probability at 2 years (probability of not being diagnosed with colorectal cancer/polyps)

***Event Probability***

*P = 1 – S(2).*

*Where P is the probability of colorectal cancer/polyp being diagnosed within 2 years of the latest FOBT date; x*_1_*Latest FOBT result; x*_2_*ex-smoker; x*_3_*current smoker; x*_4_*alcohol consumption; x*_5_*sex; x*_6_*age at FOBT; x*_7_*Number of previous negative BCSP FOBTs < 80 fL; x*_8_*Family History of GI Cancer.*

*The dataset derived for the multivariable modelling analysis had 1676 colorectal cancers and polyp diagnoses (sample population = 191,081) and considered 17 degrees of freedom in the model building process giving 98.59 events per variable. The final model had 10 degrees of freedom with an AIC of 34,050.33 and BIC 34,104.77 (N = 1676 when calculating BIC). Overall model fit was assessed using adjusted R*^*2*^*which was 0.600 (bootstrapped CI 100 reps: 0.580, 0.622) and adjusted D was 2.509. Regular R*^*2*^*was 0.602 with a D statistic of 2.519. The linear predictor from the final model had a mean of − 0.021 and a standard deviation of 1.630 (range: -446.458 to 5.048, IQR: -0.235 to 0.781).*

The final model developed for those with negative FOBT results only included; smoking status, sex, age at FOBT, previous negative BCSP FOBT results, blood test ordered by the GP and whether a patient has an IBS diagnosis. Age of FOBT was modelled using fractional polynomials and previous negative BSCP FOBT results was centred. The model is reported below in Table 3.

**Table 3:** Cox regression multivariable prediction model for patients with negative FOBTs only *n* = 187,470, 735 events

Variable	Hazard Ratio	Observed Coefficient	Bootstrapped Standard Error	z	*P* > z	[95% Confidence Intervals]	
**Smoking Status**							
Ex-smoker (reference category non-smoker)	1.238	0.214	0.078	2.75	0.006	0.061	0.366
Current smoker (reference category non-smoker)	1.499	0.405	0.148	2.74	0.006	0.116	0.694
**Sex Female (reference category male)**	0.777	−0.252	0.074	−3.42	0.001	−0.397	−0.108
**(Age/10)**^− 2^*	−	− 1581.596	639.251	−2.47	0.013	− 2834.505	− 328.687
**(Age/10)**^**− 2**^x ln(Age/10) *	−	1094.918	460.929	2.38	0.018	191.514	1998.322
Previous Negative BCSP FOBTs *	0.761	−0.272	0.066	−4.11	<0.001	−0.403	− 0.142
**GP ordered Blood Test**	1.286	0.251	0.067	3.76	<0.001	0.121	0.382
**IBS Diagnosis**	1.415	0.347	0.123	2.83	0.005	0.106	0.588

*Abbreviations: CI = confidence intervals, FOBT = faecal occult blood test (specifically guaiac). The continuous variables (Age/10)*^*−2*^*has been centred at 0.023, (Age/10)*^*− 2*^x ln(Age/10) at 0.043, Previous negative BCSP FOBTs at 0.510. A ‘*’ indicates that the variable is treated as continuous

***Survival Probability***

$$ S(2)={0.9909}^{\exp \left(0.21{x}_1+0.41{x}_2-0.25{x}_3-1582\left(\ {\left(\frac{x_4}{10}\right)}^{-2}-0.023\right)+1095\left(\ {\left(\frac{x_4}{10}\right)}^{-2}\ast \ln \left(\frac{x_4}{10}\right)-0.043\right)-0.27\left({x}_5-0.510\right)+0.251{x}_6+0.347{x}_7\right)} $$

0.9909  baseline CRC free survival at 2 years S_0_(2) (the re-estimated shrunken baseline CRC free survival at 2 years was also 0.9909 when rounded) the heuristic shrinkage factor was 0.914 where S(2) is the survival probability at 2 years (probability of not being diagnosed with colorectal cancer/polyps)

***Event Probability***

*P = 1 – S(2).*

*Where P is the probability of colorectal cancer/polyp being diagnosed within 2 years of the latest FOBT date; x*_1_*ex-smoker; x*_2_*current smoker; x*_3_*sex; x*_4_*age at FOBT; x*_5_*Previous negative BCSP FOBT; x*_6_*GP ordered blood test; x*_7_*presence of IBS.*

*There were 735 events (sample population = 187,470) and considered 16 degrees of freedom giving 45.94 events. The final model had 8 degrees of freedom with an AIC of 16,686.66 and BIC of 16,723.46 (N = 735 when calculating BIC)). Overall model fit was assessed using adjusted R*^*2*^*which was 0.066 (bootstrapped CI 100 reps: 0.046, 0.100). Regular R*^*2*^*was 0.072 (95% CI: 0.047, 0.102) with D statistic of 0.572. The linear predictor from this model had a mean of − 0.021 and a standard deviation of 0.363 (range: -1.418 to 1.206, IQR: -0.287 to 0.211).*

### Optimism adjusted model performance

Apparent performance and optimism adjusted performance for both models are reported in Table 4. For the model including both negative and positive FOBT results, Harrell’s C statistic (measure of discrimination) was 0.863 (95% CI: 0.851, 0.874). This means that the predictors used in the model correctly identify the order of survival times for pairs of patients 86% of the time. I.e. 85% out of all possible pairs of participants, the individual with higher predicted CRC free survival had a longer CRC free survival than the other participant in the selected pair (and vice versa for event probability) [30]. Van Houwelingen’s heuristic shrinkage was 0.998. For the model developed for those with negative FOBTs only, Harrell’s C statistic was 0.604 (95% CI: 0.582, 0.626). Van Houwelingen’s heuristic shrinkage was 0.914. There was minimal optimism adjustment most likely due to the large sample size.

**Table 4:** Optimism calculated performance for the C statistic, c-slope, D statistic and R^2^ for the multivariable models

Statistic	Apparent Performance	Optimism (100 bootstrap replications)	Optimism adjusted performance (apparent minus optimism)
**Model for participants with positive and negative FOBT results**			
C statistic	0.863	0.002	0.860
c-slope	1.000	0.003	0.997
D statistic	2.519	0.028	2.491
R^2^	0.602	0.005	0.597
**Model for negative FOBT patients only**			
C statistic	0.604	0.007	0.597
c-slope	1.000	0.060	0.940
D statistic	0.572	0.039	0.533
R^2^	0.072	0.010	0.062

### Calibration

**Fig. 1:**
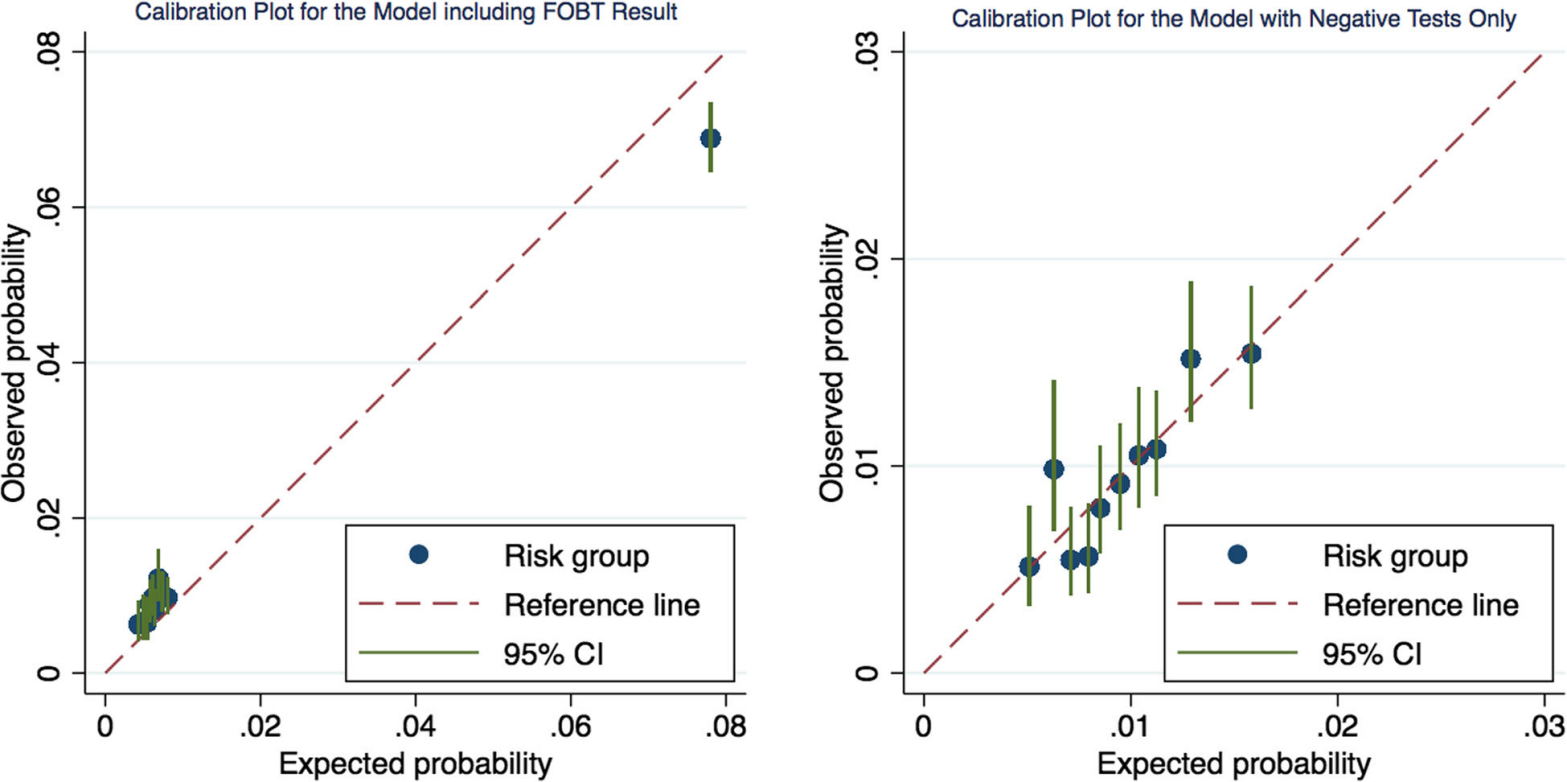
Calibration curves for the multivariable models adjusted for optimism and for deciles of risk. Left: Model for participants with both positive and negative FOBTs. Right: Model for participants with negative FOBTs only

Calibration curves for both models are presented below for deciles of risk in Fig. 1. In the model including the FOBT result, for individuals at lower risk, the model slightly underestimates the level of risk, whilst for the top risk group the model slightly overestimates the level of risk.

The separation between the risk groups gives an indication of how well the model discriminates between those with the disease and those without. The first nine groups are spaced closely together with the mean probability of the tenth group being far removed. This is most likely due to whether an individual has either a positive or negative FOBT (a particularly strong predictor). Those with a positive FOBT are designated at much higher risk. Compared to the multivariable model including the FOBT result, the spacing between groups for the model with negative tests only was more even. Risk group two in particular is being underestimated by the model but most of the groups lie close to the line of equality, indicating good calibration.

### Predicted probabilities

The equations for both models are provided in Tables 2 and 3. For participants with positive/negative FOBT results, the baseline CRC free survival at 2 years was 0.993. The mean probability of being diagnosed with CRC or polyp within 2 years was 0.013 with a standard deviation of 0.051 (Range: 0.000, 0.645). For the population with negative FOBTs only, the baseline CRC free survival for the Cox model was 0.991 at two years. The mean probability was 0.009 with standard deviation 0.0032 (Range: 0.0025, 0.0273).

### Clinical implications

The prediction model developed for those with negative FOBTs only could be used to identify additional patients for referral based on a combination of their symptoms and other demographic characteristics. A risk cut-off which represents the NICE guidelines PPV risk level of 3% in a sample of patients with complete data and 2 year follow up was investigated (*n* = 25,592). Of this population there were 449 cancers/polyps detected (5.06% FOBT positivity, 51.38% female, mean age 65.92).

**Fig. 2:**
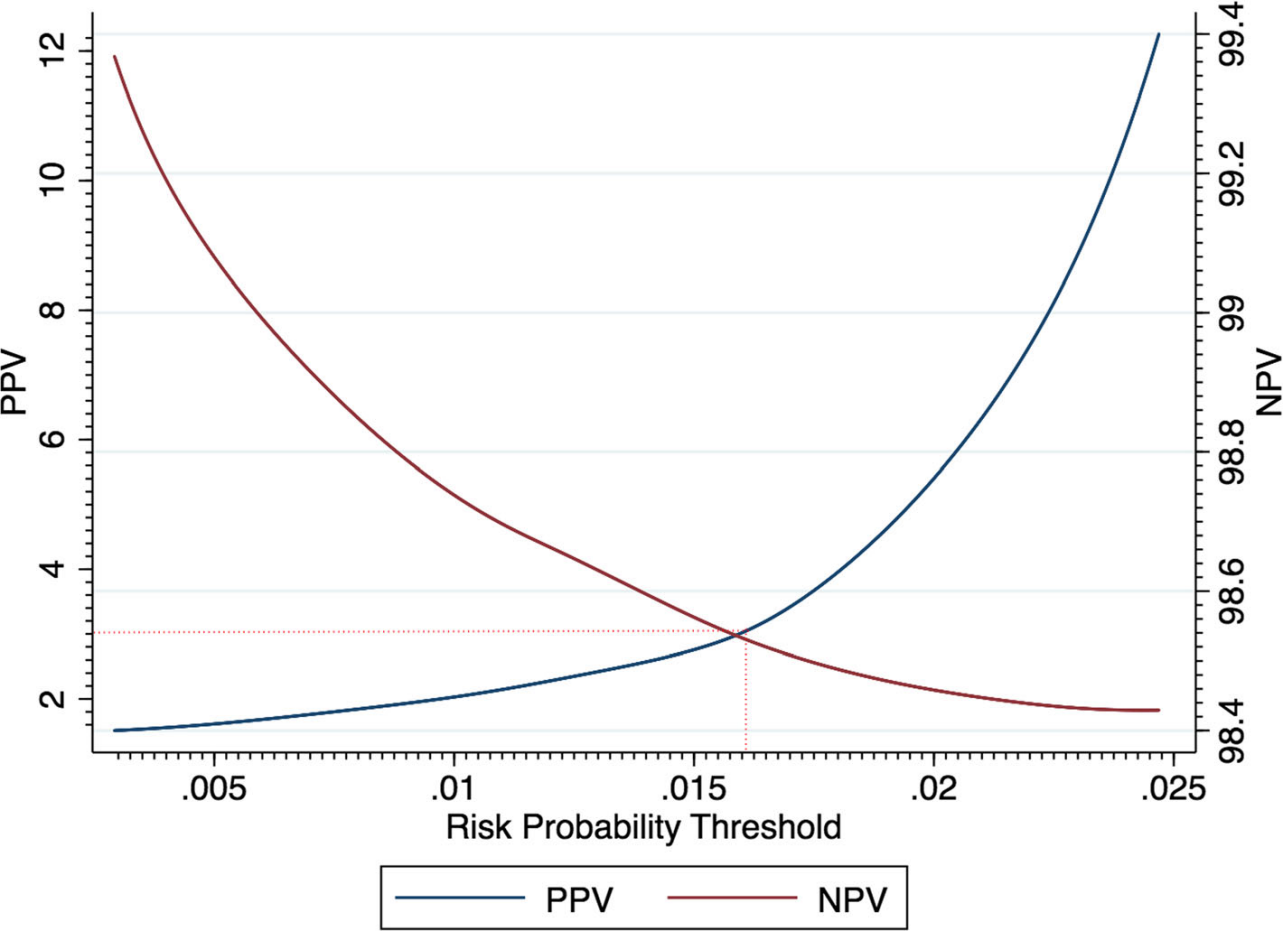
PPV and NPV for different thresholds of risk for the prediction model applied to those with negative results and with two year follow up. A PPV of 3% corresponds to a risk probability threshold of 0.0168. The plot displays the locally weighted regression lines of PPV and NPV against the risk probability determined from the model

**Fig. 3:**
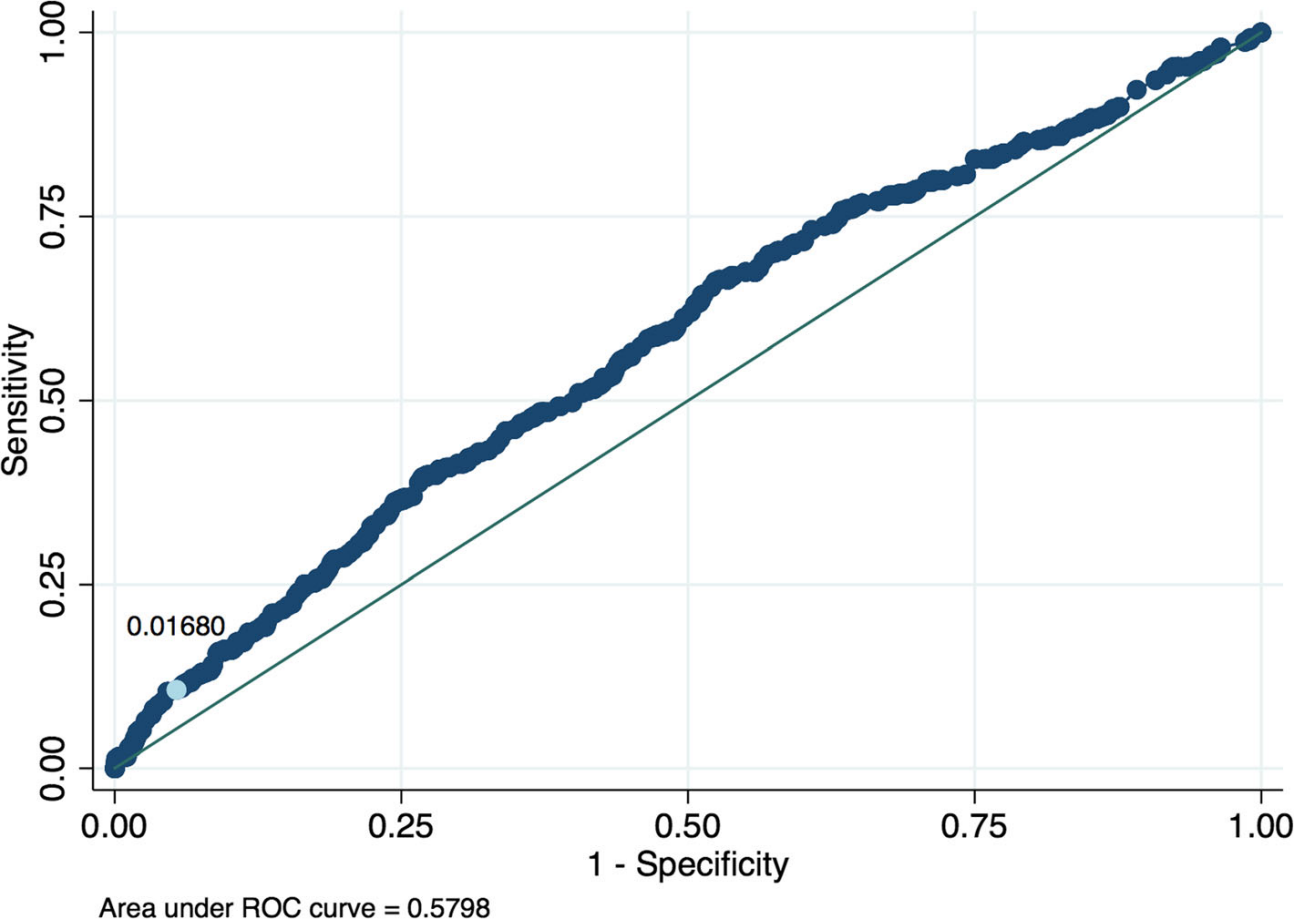
ROC curve sensitivity and specificity pairs for different thresholds of risk determined from the risk prediction model applied to those with negative results and with two year follow up (*n* = 24,297). A risk probability cutoff of 0.0168 is indicated on the curve and refers to a PPV level of 3%

For the FOBT only for this population, there was a sensitivity of 50.45% and a specificity of 95.78%. These figures are similar to estimates reported in the literature [36, 37]. A risk probability threshold for the prediction model corresponding to a NICE PPV level of 3% was determined as 0.0168 (see Fig. 2). The corresponding ROC curve for the prediction model is shown in Fig. 3.

At the probability threshold of 0.0168 the PPV of the model was 3.00%, NPV 98.51% and sensitivity 10.68% (See Table 5). For the combined strategy (either FOBT positive or risk positive if a negative FOBT result), sensitivity was 58.82% (improved from a sensitivity of 53.90% with the FOBT only) and specificity was 91.38% (this has decreased and indicates additional referrals from false positives). The number of cancers/polyps increased by 41 which is nearly a 10% increase from using the FOBT only (449 for FOBT only, 490 for FOBT positive plus risk positive for those with negative results). This is however accompanied by an increased number of referrals (1.65 times the number of FOBT only) and therefore 839 extra participants would need lower GI investigations (1295 for FOBT only to 2134 for FOBT positive and risk positive for those with negative results).

**Table 5:** 2 by 2 table for the FOBT only, model only and a combined approach of FOBT positive plus risk positive at PPV 3% level (probability: 0.0168) for those with negative results

		Diagnostic Positive: Record/diagnosis of CRC (over 2 year follow up)			Diagnostic Negative: No record/diagnosis of CRC (over 2 year follow up)
		CRC	Polyp	Total	Total
**Index Test Positive**	FOBT only *n* = 25,592	158	291	**449**	**846**
	Model Only (negative population only, *n* = 24,297)	13	28	**41**	**1288**
	Combined *n* = 25,592 (positive result is either risk positive or FOBT positive)	171	319	**490**	**2134**
**Index Test Negative**	FOBT only *n* = 25,592	165	219	**384**	**23,913**
	Model Only (negative population only, *n* = 24,297)	152	191	**343**	**22,625**
	Combined *n* = 25,592 (positive result is either risk positive or FOBT positive)	152	191	**343**	**22,625**

**FOBT Only:** Sensitivity 53.90%, Specificity 96.58%, PPV 34.67%, NPV 98.42%.

**Model Only:** Sensitivity 10.68%, Specificity 94.61%, PPV 3.00%, NPV 98.51%.

**Combined (FOBT Positive or Risk Positive):** Sensitivity 58.82%, Specificity 91.38%, PPV 18.67%, NPV 98.51%.

## Discussion

This research has assessed the availability and association of predictors for CRC in a screening population using Bowel Cancer Screening Programme results complemented with richer GP level data. Two prediction models which determine the risk of CRC/polyps were developed and included, demographics, lifestyle factors and other clinical characteristics. Risk predictors retained in the models and which might contribute to a future screening referral algorithm included; age, sex, alcohol consumption, IBS diagnosis, family history of gastrointestinal cancer, smoking status, previous negatives and whether a GP ordered a blood test 365 days before their latest screening result. Optimism adjusted performance metrics showed that the model including the FOBT result had good discrimination (C statistic: 0.860) and was well calibrated.

The model for participants with negative results had a discrimination of 0.597. The performance of this model could be improved with the inclusion of further predictors or ideally the newer FIT could be combined with these risk factors so that if an individual is under a particular cut-off, this could be adjusted based on the presence of further predictors. As datasets become more diverse and multifaceted, machine-learning approaches may be better placed to deal with more complex data. Calculating individual risk using prediction models can help referral decisions as well as patients and screening practitioners make a more informed choice.

Although the risk prediction model developed for BCSP FOBT negative patients led to an increase in the number of cancers detected in a combined approach of FOBT positive and risk positive, this also caused an increased number of individuals undergoing GI associated investigations. Depending on available resources, this model would therefore not be clinically useful in its current iteration. A more nuanced algorithm combining the newly available quantitative FIT screening test result would allow a spectrum of risk to be combined with other predictors as the concentration of haemoglobin detected has shown to be associated with the level of risk. This research has however identified several potential predictors which could be combined with the FIT by exploiting the interface between the screening database and primary care records.

The models developed and predictors selected build on the findings from other models which have been developed for use in a primary care population. The discrimination of these models were comparable to the results obtained in the current study for the model combining the FOBT (AUC ROC of 0.83 for a logistic regression model and 0.89–0.91 C-statistic for Cox regression models respectively) [13, 15].

To our knowledge this is the first instance of exploiting a primary care dataset for a screening population using the electronic notifications sent from the BCSS to primary care. Predictor variables retained in the final model developed by Hippisley-Cox et al. [15] included, age, family history of gastrointestinal cancer, anaemia, rectal bleeding, abdominal pain, appetite loss and weight loss (alcohol status and recent change in bowel habit were also significant for males). Since this model was developed for primary care, red flag symptoms such as abdominal pain and rectal bleeding were included. The AUC ROC was 0.89 for females and 0.91 in males in the validation sets. This has higher performance than the current study (C statistic: 0.860) but included strong red flag predictors and was developed for use in a different setting (primary care).

Although blood test results were available to combine in the prediction models, they were not available for all participants (recorded around 45% for haemoglobin, MCV, platelet count). This is due to a reflection of the underlying clinical process where a blood test is carried out if a GP suspects disease. The univariable associations do however show the potential of using blood test results in a future prediction algorithm, ideally taking into account multiple measures over time and their variability.

Other studies have shown the merit of using blood test results combined with screening tests [8, 39, 40]. For isntance, a study using the THIN database and the Maccabi Healthcare Services (an Israeli dataset) combined blood measures, sex and age in a machine learning model (random forest model) to determine which individuals were at increased risk for CRC [8]. This model gave an AUC of 0.82. By combining the FOBT with the lab results and comparing it to the gFOBT alone, the model identified 48% more CRC cases [8]. The added effect of lab data may help to reduce false negatives from the screening test since FOBTs may fail to identify intermittent bleeding or low level bleeding. Inclusion of longitudinal laboratory test results could help to predict future disease.

Strengths of this study include the use of data originating from different healthcare systems; BCSP results complemented with richer GP data not usually available to contribute to referral decisions and prediction algorithms. Combining data from multiple sources enables a clearer and fuller picture of patient profiles using the primary care and screening database interface.

Further strengths include the sample size of the BCSP cohort and the range of predictors available from GP records assessed for completeness and association. The methods used to derive these data were thorough and subject to review by two people. Internal validation was used to adjust model performance measures for optimism. There was minimal optimism adjustment most likely due to the size of the dataset.

Missing data was a limitation of this study, however this was limited mostly by the continuous variable alcohol consumption which was still recorded in nearly 80% of cases. Other variables such as BMI (95.85%) and smoking status (99.44%) were highly complete and other conditions/symptoms were recorded if observed. Since missing data can lead to bias in parameter estimates and reduce sample size and generalizability, multiple imputation was considered which leads to more accurate standard errors and *p*-values compared to other missing data methods. The missing data mechanism for the majority of these predictors however would be ‘Missing Not At Random’ (MNAR) [41]. Individuals who had a blood test result for example were more likely to have this investigation based on suggestive symptoms of a particular underlying disease.

There is differential verification of cancer in this dataset because it is real world data. Participants with positive FOBT results would be more likely to be referred for colonoscopy and receive quicker diagnosis compared to those with negative FOBT results which would rely more on follow up (ascertainment bias). Therefore, the model may overestimate the predictive power of FOBT and other variables used in the current pathway to determine whether to refer for colonoscopy, and underestimate the predictive power of those variables not used in the referral pathway. This is a limitation of using routine data. Furthermore, the data does not include granularity on the different diagnostic types used in a secondary care setting; this may result in additional verification bias. Linkage to HES (Hospital Episode Statistics) could provide this higher level of detail.

Due to the dichotomous nature of the gFOBT and due to the continuing replacement of this test worldwide a similar approach should be investigated for the newer quantitative FIT where the concentration has been shown to relate to the level of risk [42]. An approach combining FIT has shown promise in recent research [6, 7, 43, 44]. At the time of data collection, FIT results had not been populated onto GP records (there is also not currently a feature which records the numerical result) therefore the gFOBT was used as the screening test for this research.

The prediction models or identified variables from this study could be considered for use at various points along the CRC screening pathway. A model including the test result and other clinical features could be used to decide which participants are at highest risk for referral using a probability threshold. The predictors identified from this study could also be considered for inclusion in a model which decides a screening interval (surveillance) for an individual determined from a baseline risk or first screening result. Alternatively the predictors could be used to identify a starting population who would benefit most from screening.

There is capacity to draw out this additional information from the NHS Spine (with data originating from GP records) to the BCSS. The factors shown in this study to be predictive of CRC could be considered in the future to combine with the screening test to identify those at highest risk and who would benefit most from limited colonoscopy services. This research shows the potential of linking datasets for improved healthcare which is a key directive of initiatives such as the NHS Long Term Plan, Connecting Care and the establishment of research data hubs [45].

## Conclusions

This research has identified several potential predictors for CRC in a screening population by exploiting the interface between the screening database and primary care records. These predictors can be considered in a refined risk prediction model combining the newer quantitative FIT for bowel cancer screening. Additional data could be drawn onto the screening database to contribute to a referral algorithm to improve colonoscopy use and to benefit those at highest risk of CRC.

## Supplementary information

**Additional file 1: Table S1.** Variables assessed for univariable and multivariable analysis

**Additional file 2: Figure S1.** Study flow diagram for data extraction

**Additional file 3: Figure S2.** Study flow diagram for data analysis

**Additional file 4: Table S2.** 2 by 2 table of colorectal cancer/polyp diagnosis by guaiac faecal occult blood test (gFOBT) result for participants with 2 years of follow up.

**Additional file 5: Table S3.** Cancer/polyp detection rates for participants with and without laboratory results (haemoglobin concentration, MCV and platelet count) *N* = 292,059.

**Additional file 6: Table S4.** Additional variables assessed for completeness and univariable associations with colorectal cancer and polyps.

## Data Availability

The data that support the findings of this study are available from The Health Improvement Network (THIN) available from IQVIA but restrictions apply to the availability of these data, which were used under license for the current study, and so are not publicly available. Data are however available from IQVIA and subject to approval by their Scientific Review Committee [https://www.iqvia.com/locations/uk-and-ireland/thin-hes-data]. The Read code and drug code lists will be made available on ClinicalCodes.org repository [https://clinicalcodes.rss.mhs.man.ac.uk/]. Further data extraction methods (Additional Health Data) are available on reasonable request from the authors.

## Abbreviations

AMR: Acceptable Mortality Reporting
AUC: Area Under the Curve
BCSP: The Bowel Cancer Screening Programme
BCSS: Bowel Cancer Screening System
BMI: Body Mass Index
BNF: British National Formulary
CRC: Colorectal Cancers
FAP: familial adenomatous polyposis
FIT: Faecal Immunochemical Test
FOBT: Faecal Occult Blood Test
gFOBT: Guaiac Faecal Occult Blood Test
GI: Gastro Intestinal
GP: General Practitioner
Hb: Haemoglobin
HNPCC: hereditary nonpolyposis colorectal cancer
HR: Hazard Ratio
IBS: Irritable Bowel Syndrome
MCV: Mean Cell Volume
MFP: Multivariable Fractional Polynomial
MNAR: Missing Not At Random
NHS: National Health Service
NICE: National Institute for Health and Care Excellence
NPV: Negative Predictive Value
PMIP: Pathology Messaging Implementation Programme
PPV: Positive Predictive Value
QOF: Quality Outcomes Framework
RECORD: REporting of studies Conducted using Observational Routinely-collected Data
ROC: Receiver Operating Characteristic
SNOMED CT: Systematized Nomenclature of Medicine - Clinical Terms
THIN: The Health Improvement Network
TRIPOD: Transparent Reporting of a multivariable prediction model for Individual Prognosis Or Diagnosis
