# Supplementary material for: The use of electronic healthcare records for colorectal cancer screening referral decisions and risk prediction model development
Source: BMC Gastroenterol. 2020 Mar 25;20:78. doi: 10.1186/s12876-020-01206-1 (PMC7093989; doi:10.1186/s12876-020-01206-1)
Supplement: Supplementary file 1 — Additional file 1: Table S1. Variables assessed for univariable and multivariable analysis [file 12876_2020_1206_MOESM1_ESM.docx]

**Figure S1** Study flow diagram for data extraction

**THIN Database**744 practices
16,458,973 patients

**Restrict practices to England**497 practices
12,063,499 patients

**Restrict if AEB date is missing (i.e. the practice does not receive electronic screening notifications)**361 practices
9,208,166 patients

**Restrict if Registration date is missing in patient records**9,208,166 patients

**Patient does not have a ‘patflag’ A or C (Marker of data quality in THIN)**
7,796,029
**Patient record does not have a ‘regstat’ of 01, 02, 05, 99
(Marker of data quality in THIN)**7,796,029

**Restrict patients if patient start date is greater or equal to patient end date**646,807 patients

**Restrict to patients with a Townsend Score**631,747 patients

**Restrict to patients with a BCSP notification during period of interest**445,533 BCSP notifications included
360 practices
299,315 distinct patients

BCSP FOB test positive: 8,057
BCSP test negative: 429,014
BCSP FOB testing kit spoilt: 42
BCSP FOB test incomplete participation: 8,420

**Exclude those with a high risk condition**169 FAP records

299,203 distinct patients
360 practices

**Exclude those with a previous CRC diagnosis**2143 CRC records before the BCSP FOBT date (bcsp_fobt_evntdate)

**297,735 distinct patients**
360 practices
