# Supplementary material for: The use of electronic healthcare records for colorectal cancer screening referral decisions and risk prediction model development
Source: BMC Gastroenterol. 2020 Mar 25;20:78. doi: 10.1186/s12876-020-01206-1 (PMC7093989; doi:10.1186/s12876-020-01206-1)
Supplement: Supplementary file 2 — Additional file 2: Figure S1. Study flow diagram for data extraction [file 12876_2020_1206_MOESM2_ESM.docx]

**Figure S2**. Study flow diagram for data analysis

**Patients latest BCSP Result (n=297,735) this included incomplete participation and spoilt kits**BCSP FOB test positive: 6,338
 BCSP FOB test negative: 285,432
 BCSP FOB testing kit spoilt: 31
BCSP FOB test incomplete participation: 5,934

**Patients with positive/negative test (latest result included only)
(n = 292,059) 360 practices**6,362 positive
285,697 negative

**Patients with more than one BCSP round electronic notification (n=297,735) this include incomplete participation and spoilt kits**179,158 patients have 1 notification
92,885 have 2 notifications
24,498 have 3 notifications
1,172 have 4 notifications
21 have 5 notifications
1 has 6 notifications

**Number of BCSP round electronic notifications (n=443,221) this includes incomplete participation and spoilt kits**BCSP FOB test positive: 7,964
BCSP FOB test negative: 426,866
BCSP FOB testing kit spoilt: 42
BCSP FOB test incomplete participation: 8,349
