# Supplementary material for: The use of electronic healthcare records for colorectal cancer screening referral decisions and risk prediction model development
Source: BMC Gastroenterol. 2020 Mar 25;20:78. doi: 10.1186/s12876-020-01206-1 (PMC7093989; doi:10.1186/s12876-020-01206-1)
Supplement: Supplementary file 3 — Additional file 3: Figure S2. Study flow diagram for data analysis [file 12876_2020_1206_MOESM3_ESM.docx]

**Table S1** Variables assessed for univariable and multivariable analysis

| **Variable Type** | **Variables Considered for Multivariable Analyses** | **Additional Variables Assessed in Univariable Analyses** |
| --- | --- | --- |
| **Screening Utilisation** | Latest FOBT result, previous negative FOBT result from BCSP, previous positive FOBT result from BCSP.  All previous BCSP FOBT results prior to index date. Previous negative and previous positive FOBTs were coded as continuous variables. | Previous Screening variable was created as a variable based on whether an individual has one or more previously recorded positive or negative FOBT result. Binary variable, if ever previously screened.  Previous polyp if ever recorded (binary variable) latest date prior to index date.  A FOBT performed in primary care up to 365 days prior to the index date. Coded as a binary variable |
| **Laboratory test results** | Blood test result up to 365 days before the index date. Coded as a binary variable and based on the presence of either a result for Haemoglobin, MCV, platelet count or ferritin. | Haemoglobin category, MCV category, platelet count category, ferritin category. Latest record up to 365 days prior to the index date. (Coded using clinically derived cut-offs as binary variables)  The percentage change between the last two recorded values before the index date for all blood tests listed above to capture longitudinal trends.  Investigated coded continuously, as % change and using a clinically derived cut-off.  All lab results recorded for 2 years prior to the index date were also extracted so they could be investigated longitudinally. |
| **Co-morbidities and previous diagnoses** | Diabetes, IBS coded as binary variables. | Crohn’s disease diagnosis, ulcerative colitis, diverticulitis all if ever recorded prior to index date.  Venous thromboembolism date of most recent up to 365 days before.  All coded as binary variables. |
| **Demographic Factors** | Sex, age at FOBT  Age was coded continuously, sex as a binary variable. | Ethnic Group (last recorded entry) – factor with 5 groupings (white at baseline)  Blood group (last recorded entry) with 4 groups, Type A at baseline. |
| **Lifestyle and anthropometric factors** | Smoking status, BMI, alcohol consumption.  Last recorded entry prior to index date for alcohol consumption, smoking status.  Smoking status coded as a factor with 3 levels, non-smoker at baseline.  Alcohol consumption (number of units consumed a week) and BMI coded as continuous variables. | Height, weight, weight % change between two most recent readings,  Latest record before the index date.  BMI % change between two most recent readings. |
| **Symptoms** | - | Antispasmodic drug prescription Anti-motility drug prescription Laxative drug  Weight loss, abdominal pain combined with antispasmodic prescription, constipation, diarrhoea, change in bowel habit, loss of appetite, tiredness, flatulence, rectal bleeding, abdominal mass.  (Date of the most recent prior to the index date up to 365 days before)  Coded as binary variables. |
| **Additional** **Factors** | Townsend quintile (last recorded prior to index date), family history of gastrointestinal cancer (if ever recorded before index date).  Townsend quintile coded as a factor with 5 levels with 1 at baseline. Family history coded as a binary variable. | Urban rural split into 4 groups (last recorded prior to index date)  Urban rural Town & Fringe – less sparse at baseline |
| Abbreviations: FOBT = BCSP = bowel cancer screening programme, faecal occult blood test, BMI = body mass index, MCV = mean cell volume, IBS = irritable bowel syndrome. | | |
