# Supplementary material for: The use of electronic healthcare records for colorectal cancer screening referral decisions and risk prediction model development
Source: BMC Gastroenterol. 2020 Mar 25;20:78. doi: 10.1186/s12876-020-01206-1 (PMC7093989; doi:10.1186/s12876-020-01206-1)
Supplement: Supplementary file 4 — Additional file 4: Table S2. 2 by 2 table of colorectal cancer/polyp diagnosis by guaiac faecal occult blood test (gFOBT) result for participants with 2 years of follow up. [file 12876_2020_1206_MOESM4_ESM.docx]

**Table S2** 2 by 2 table of colorectal cancer/polyp diagnosis by guaiac faecal occult blood test (gFOBT) result for participants with 2 years of follow up.

| **gFOBT result** | **Cancer /Polyp Diagnosis Positive** | **Cancer /Polyp Diagnosis Negative** | **Total** |
| --- | --- | --- | --- |
| **Positive** | 195 colorectal cancers 354 polyps  =549 | 1,084 | 1633 |
| **Negative** | 203 colorectal cancers 320 polyps  =523 | 28,031 | 28,554 |
| **Total** | 1,072 | 29,115 | 30,187 |
| N=30,187 Positivity: 5.41%, Sensitivity 51.21%, Specificity: 96.28% Participants were 51.88% female with a mean age of 66.00 years. | | | |
