# Supplementary material for: The use of electronic healthcare records for colorectal cancer screening referral decisions and risk prediction model development
Source: BMC Gastroenterol. 2020 Mar 25;20:78. doi: 10.1186/s12876-020-01206-1 (PMC7093989; doi:10.1186/s12876-020-01206-1)
Supplement: Supplementary file 5 — Additional file 5: Table S3. Cancer/polyp detection rates for participants with and without laboratory results (haemoglobin concentration, MCV and platelet count) N = 292,059. [file 12876_2020_1206_MOESM5_ESM.docx]

**Table S3** Cancer/polyp detection rates for participants with and without laboratory results (haemoglobin concentration, MCV and platelet count) N= 292,059.

|  | **Haemoglobin Concentration** | | **MCV** | | **Platelet Count** | |
| --- | --- | --- | --- | --- | --- | --- |
|  | *Without Record* | *With Record* | *Without Record* | *With Record* | *Without Record* | *With Record* |
| **Cancer/Polyp** | 1345 | 1544 | 1,352 | 1,537 | 1,349 | 1,540 |
| **No Cancer/Polyp** | 160,718 | 128,452 | 161,226 | 127,944 | 161,025 | 128,145 |
| **Total** | 162,063 | 129,996 | 162,578 | 129,481 | 162,374 | 129,685 |
| **Cancer Detection Rate (%)** | 0.830 | 1.188 | 0.832 | 1.187 | 0.831 | 1.187 |
