# Supplementary material for: The use of electronic healthcare records for colorectal cancer screening referral decisions and risk prediction model development
Source: BMC Gastroenterol. 2020 Mar 25;20:78. doi: 10.1186/s12876-020-01206-1 (PMC7093989; doi:10.1186/s12876-020-01206-1)
Supplement: Supplementary file 6 — Additional file 6: Table S4. Additional variables assessed for completeness and univariable associations with colorectal cancer and polyps. [file 12876_2020_1206_MOESM6_ESM.docx]

**Table S4**: Additional variables assessed for completeness and univariable associations with colorectal cancer and polyps

| **Variable** | **Percentage with this  variable recorded  (N = 292,059)** | | **Prevalence of variable (%)** | | **Hazard Ratio  (95% Confidence Interval)** | | **Standard  Error** | **P>z** |
| --- | --- | --- | --- | --- | --- | --- | --- | --- |
| **Sociodemographic characteristics** |  |  |  |  |  |  |  |  |
| **Ethnic Group** | 54.76 | (159,926/292,059) |  |  |  |  |  |  |
| White (baseline) | 51.97 | (151,782/292,059) | 94.91 | (151,782/159,926) |  |  |  |  |
| Asian | 1.56 | (4,557/292,059) | 2.85 | (4,557/159,926) | 0.996 | (0.741-1.340) | 0.151 | 0.980 |
| Black | 0.70 | (2,047/292,059) | 1.28 | (2,047/159,926) | 0.786 | (0.480-1.286) | 0.197 | 0.337 |
| Mixed | 0.21 | (610/292,059) | 0.38 | (610/159,926) | 1.363 | (0.680-2.731) | 0.483 | 0.382 |
| Other | 0.32 | (930/292,059) | 0.58 | (930/159,926) | 0.565 | (0.235-1.358) | 0.253 | 0.202 |
|  |  |  |  |  |  |  |  |  |
| **Townsend quintile** | 98.11 | (286,530/292,059) |  |  |  |  |  |  |
| 1 (least deprived) | 36.16 | (105,616/292,059) | 36.86 | (105,616/286,530) |  |  |  |  |
| 2 | 25.44 | (74,295/292,059) | 25.93 | (74,295/286,530) | 1.065 | (0.968-1.172) | 0.052 | 0.199 |
| 3 | 18.71 | (54,637/292,059) | 19.07 | (54,637/286,530) | 1.041 | (0.937-1.157) | 0.056 | 0.455 |
| 4 | 11.84 | (34,584/292,059) | 12.07 | (34,584/286,530) | 1.162 | (1.031-1.308) | 0.070 | 0.014* |
| 5 | 5.96 | (17,398/292,059) | 6.07 | (17,398/286,530) | 1.387 | (1.200-1.603) | 0.102 | 0.000* |
| **Weight related** |  |  |  |  |  |  |  |  |
| Height | 96.06 | (280,563/292,059) | Mean 1.68 (SD 0.10) |  | 4.390 | (3.001-6.420) | 0.852 | 0.000* |
| Weight | 96.74 | (282,550/292,059 | Mean 78.08 (SD 16.53) |  | 1.012 | (1.010-1.014) | 0.001 | 0.000* |
| BMI (% change)† | 30.98 | (90,484/292,059) | Mean -0.08 (SD 4.62) |  | 0.994 | (0.982-1.007) | 0.006 | 0.385 |
| Weight % change† | 31.07 | (90,729/292,059) | Mean -0.07 SD 4.78 |  | 0.994 | (0.981-1.006) | 0.006 | 0.324 |
|  |  |  |  |  |  |  |  |  |
| **Weight % change (as category)** | 31.07 | (90,729/292,059) | - | - | - | - | - | - |
| <5% at baseline | 28.19 | (82,339/292,059) | 90.75% (<5%) | (82,339/90,729) | - | (---) | - | - |
| 5-9.9 | 2.32 | (6,766/292,059) | 7.46% (5-9.9%) | (6,766/90,729) | 1.060 | (0.857-1.310) | 0.115 | 0.593 |
| =>10 | 0.56 | (1,624/292,059) | 1.79% (=>10) | (1,624/90,729) | 0.948 | (0.609-1.476) | 0.214 | 0.814 |
| **Laboratory test results** |  |  |  |  |  |  |  |  |
| Hb % change† | 33.87 | (98,931/292,059) | Mean 0.32 (SD 6.57) |  | 0.997 | (0.988-1.006) | 0.005 | 0.449 |
| Mean Cell Volume % change† | 33.66 | (98,304/292,059) | Mean 0.07 (SD 2.80) |  | 0.981 | (0.961-1.001) | 0.010 | 0.064 |
| Ferritin % change† | 3.52 | (10,287/292,059) | Mean 43.70 (SD 334.31) |  | 1.000 | (0.999-1.000) | 0.000 | 0.923 |
| Platelet Count % change† | 33.75 | (98,566/292,059) | Mean 0.96 SD (40.24) |  | 1.000 | (0.998-1.002) | 0.001 | 0.901 |
| **Blood Group** | 2.55 | (7,444/292,059) |  | - | - | - | - | - |
| A (baseline) | 1.07 | (3,116/292,059) | 41.86% | (3,116/7,444) | - | (---) | - | - |
| AB | 0.09 | (249/292,059) | 3.34% | (249 /7,444) | 0.443 | (0.060-3.249) | 0.450 | 0.423 |
| B | 0.25 | (739/292,059) | 9.93% | (739/7,444) | 0.708 | (0.274-1.829) | 0.343 | 0.475 |
| O | 1.14 | (3,340/292,059) | 44.87% | (3,340/7,444) | 0.975 | (0.585-1.625) | 0.254 | 0.923 |
| * Significant at the p value of 0.05  † Difference between last two records  BMI = body mass index, MCV = mean cell volume, Hb = haemoglobin concentration | | | | | | | | |
